# The impact of aging on locomotor recovery in preclinical models of traumatic spinal cord injury: a systematic review

**DOI:** 10.3389/fneur.2026.1745250

**Published:** 2026-06-01

**Authors:** Aniqah I. Bhatti, Zhikai Li, Natalia Jagodzinska, Yuhan Guo, Faheem I. Bhatti, Zainab I. Bhatti, Jamie F. M. Brannigan, Benjamin M. Davies, Mark R. Kotter, Oliver D. Mowforth

**Affiliations:** 1 School of Clinical Medicine, University of Cambridge, Cambridge, United Kingdom; 2 University of Nottingham Medical School, Nottingham, United Kingdom; 3 Nuffield Department of Clinical Neurosciences, University of Oxford, Oxford, United Kingdom; 4 Division of Neurosurgery, Department of Clinical Neurosciences, University of Cambridge, Cambridge, United Kingdom

**Keywords:** age, locomotion recovery, preclinical study, spinal cord injury, traumatic spinal cord injuries

## Abstract

**Introduction:**

Aging is known to influence recovery following spinal cord injury (SCI) however its specific impact on locomotor outcomes remains underexplored. Understanding these age-related differences is critical for developing targeted rehabilitation strategies and improving the translational relevance of SCI research. This systematic review aimed to evaluate the effect of aging on locomotor recovery in animal models of traumatic SCI.

**Methods:**

A systematic search of MEDLINE and Embase was conducted to identify studies assessing the impact of aging on post-SCI locomotor outcomes. Inclusion criteria encompassed preclinical studies comparing locomotor recovery between young and aged animals following SCI. Extracted data included sample characteristics, SCI model, locomotor outcome measures, timing of evaluation, and key findings. Risk of bias was assessed using the SYRCLE checklist.

**Results:**

Of 3,118 unique records screened, nine studies met inclusion criteria. Included animals were grouped into young (mean 2.5 months), intermediate (mean 11.4 months), and aged (mean 21.5 months) categories, with individual ages ranging from 4 weeks to 28 months. Six studies used rats and three studies used mice. In total, more than 340 animals were studied. SCI models included cord contusion (6/9, 66.7%), hemisection (2/9, 22.2%), and clip compression (1/9, 11.1%). Seven (7/9, 77.8%) studies employed the Basso, Beattie, Bresnahan (BBB) locomotor score as the primary outcome measure. Older animals demonstrated significantly lower BBB scores post-injury compared to younger counterparts in 100% (7/7) of studies using this outcome. Other measures of locomotor outcomes included the Basso Mouse Scale, CatWalk, and Digigait. Notably, one study reported that pre-injury and post-injury exercise improved locomotor recovery in aged rats to levels comparable with young rats.

**Conclusion:**

Aging is associated with poorer locomotor recovery following traumatic SCI in preclinical models. These findings underscore the importance of age as a biological variable in SCI research and suggest that rehabilitative interventions, such as exercise, may have potential to mitigate age-related deficits. Future studies should seek to define the mechanistic pathways underlying impaired recovery with age and evaluate targeted therapies that enhance neuroplasticity and functional recovery.

**Systematic review registration:**

https://www.crd.york.ac.uk/PROSPERO/view/CRD42022230021.

## Introduction

Greater than 100,000 individuals in the United Kingdom live with spinal cord injury (SCI), with approximately 4,500 new cases annually (1). SCI encompasses various forms including acute traumatic injury, degenerative myelopathy and spinal cord ischaemia. The age demographic of patients diagnosed with SCI is broad. In traumatic SCI, which is the most studied type in animal models, there is a bimodal age distribution predominantly affecting both young adults (20–29 years) and older adults (≥60 years) (2–4).

Traumatic SCI management is currently divided into acute and chronic phases, in which various factors such as the severity and type of injury and the degree of neurological impairment guide patient management (5). In the acute phase, management focuses on preventing secondary neuronal injury through measures including spinal immobilization and blood pressure augmentation (5). Timely surgical decompression may also be required (6). Management in the chronic phase includes extensive rehabilitation involving a multidisciplinary team of physiotherapists, occupational therapists, psychologists, social support workers and rehabilitation medicine doctors (7, 8). Many SCI patients experience limited recovery and live with long-term disability (7, 9).

Although good clinical decision-making will consider comorbidities and frailty, current SCI management guidelines do not explicitly make age-stratified recommendations. However, studies have identified age an important prognostic factor influencing recovery following SCI. Survival rates within the 1 year following traumatic SCI are significantly lower in older individuals (10). In addition, long-term recovery demonstrates a clear age-influenced trajectory, with increasing age associated with reduced recovery of independence in activities of daily living (11). Age also prolongs physiological recovery (12); elderly patients are known to have more surgical complications and longer duration of inpatient stay following spinal surgery (13, 14). At a cellular level, the effects of age on SCI are mediated by an age-related decline in neuroplasticity, altered immunological responses and impaired regenerative capacity (15).

Preclinical models offer a convenient and controlled experimental platform to investigate the impact of age on SCI recovery (15–18). In animal models, locomotor recovery after SCI has been quantified using a range of scoring systems such as Basso, Beattie, Bresnahan (BBB) scale and Basso Mouse Scale (BMS) (19–21). Whilst there is currently a lack of consensus on age categorization thresholds, recent studies suggest that aged rodents exhibit greater SCI lesion volumes, increased inflammation, and reduced neurotrophic support compared to their younger counterparts (15, 22, 23). These findings provide potential translational insights to inform research on age-related differences in human SCI recovery and the potential to investigate the use of age in individualizing management of SCI.

This systematic review aimed to evaluate the effect of age at time of injury on locomotor recovery in preclinical models of SCI, including exploring the underlying molecular mechanisms, assessing translational relevance, and identifying therapeutic targets that may improve outcomes in older populations.

## Methods

### Study design and registration

A systematic review was conducted following the Preferred Reporting Items for Systematic Reviews and Meta-Analysis (PRISMA) 2020 checklist (24). The review protocol was registered on the PROSPERO international prospective register of systematic reviews (CRD42022230021).

### Search strategy and study selection

A systematic search was performed in MEDLINE and Embase databases from inception to May 3, 2025. MEDLINE and Embase searches were performed using the Ovid platform (Ovid Technologies, New York, NY, United States).

Scoping searches were performed to refine the search terms. The final search strategy is presented in Supplementary Table 1. Reference lists of included studies were also screened to identify additional eligible records. No automated search limits were used to maximize sensitivity.

Studies were eligible for inclusion if they were primary research studies, employing animal models of spinal cord injury, comparing locomotor recovery between at least two age groups, and were available in English. Eligible SCI models included contusion, crush, hemisection, and clip–compression. Studies were required to report locomotor outcomes using validated behavioral assessments such as the Basso, Beattie and Bresnahan (BBB) score, Basso Mouse Scale (BMS), CatWalk, Digigait, or footprint analysis. Studies were excluded if they involved human participants, were case reports, conference abstracts, opinion pieces, corrections, systematic reviews, or meta–analyses, or if they assessed only non–locomotor outcomes such as pain, histology, or autonomic function.

Duplicates records were removed using Rayyan (Rayyan Systems, United States) (25). All screening was performed by two reviewers (AB/FB/ZB/ZL/YG). Disagreements were resolved through discussion between the reviewers until agreement was reached.

### Data extraction

Data was extracted manually in Microsoft Excel (Version 16.100, Microsoft 365) using a piloted extraction form by one of the authors (AB) and independently verified by a second author (ZL). Data extracted from each study were: author, year of publication, country of experiment, sample characteristics (e.g. species, strain, age, sex, weight), intervention (injury model and type), results of any locomotor assessment, and the nature of any statistical analysis performed.

### Risk of bias assessment

The SYRCLE (Systematic Review Center for Laboratory Animal Experimentation) tool (26) was used to evaluate the risk of bias of included studies (Supplementary Table 2).

### Synthesis methods

Due to the diverse range of injury models, interventions and outcomes, meta-analysis was not possible, and a narrative synthesis was conducted using the Synthesis Without Meta-Analysis (SWiM) reporting guideline. A checklist of adherence is provided in Supplementary Table 3. Studies were grouped based on locomotor outcome measures (27).

## Results

### Study selection

**Figure 1:**
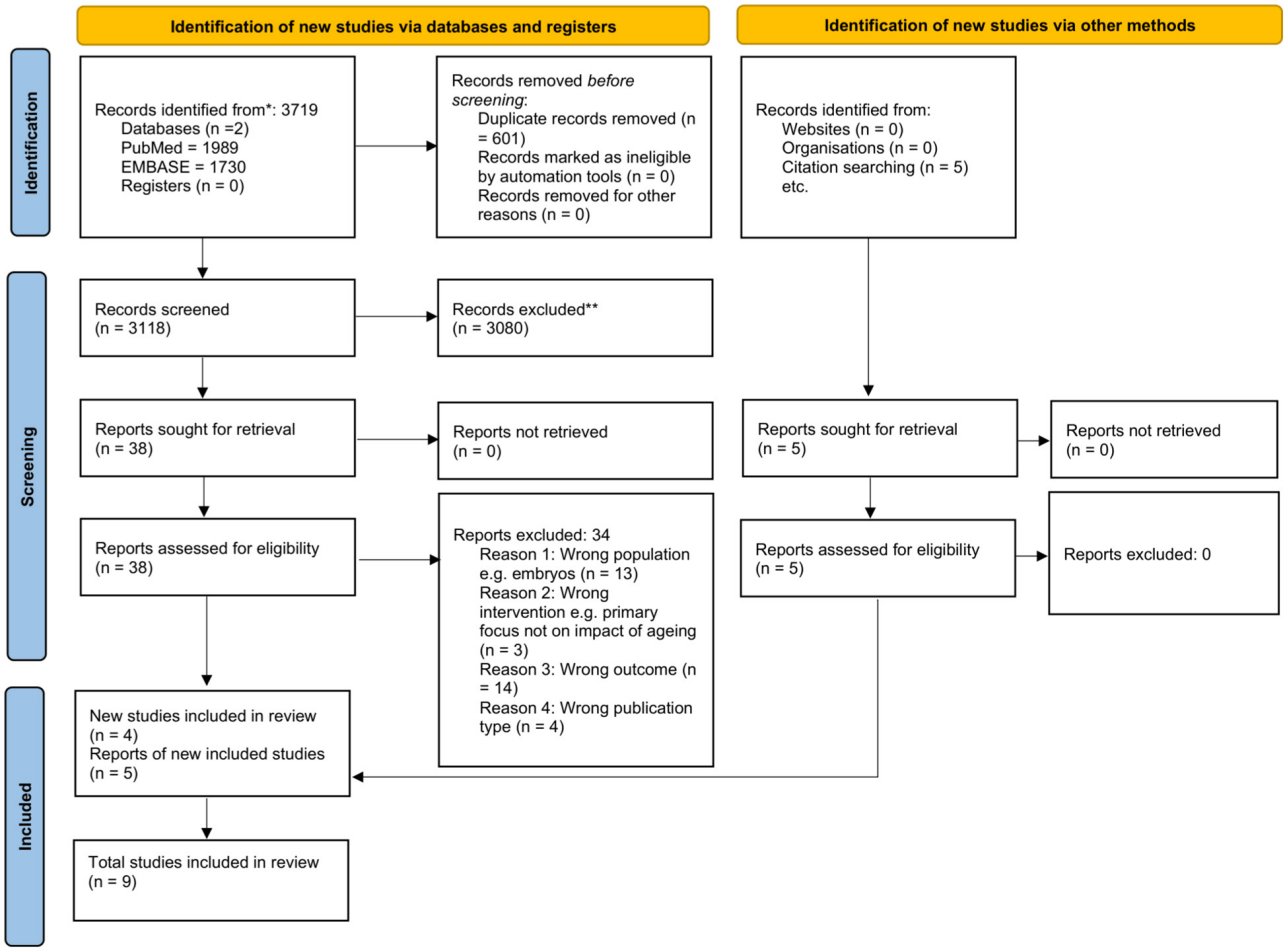
PRISMA diagram of study selection (24). A total of nine studies were included in the final analysis.

The search generated 3,719 results. After removing 601 duplicates, 3,118 unique studies remained. Abstract screening identified 38 eligible studies, and following full-text screening, 34 studies were excluded for reasons outlined in Figure 1, leaving four studies which were included in the final analysis. Five additional relevant studies (28–32) were identified from the reference lists of included studies.

### Study characteristics

Of the nine included studies, six (6/9, 66.7%) studies used rat models, while three (3/9, 33.3%) used mouse models (Table 1). Sprague-Dawley rats were used in five (5/9, 55.6%) studies and Fisher 344/Brown Norwegian F1 hybrid rats were used in one (1/9, 11.1%) study. C57BL/6 mice were used in two (2/9, 22.2%) studies and BALB/c mice were used in one (1/9, 11.1%) study. Female animals were used in four (4/9, 44.4%) studies, whilst male animals were used in three (3/9, 33.3%) studies. Two (2/9, 22.2%) studies did not specify the sex of animals used. More than 340 animals were included, across all studies (Table 2). Study characteristics are detailed in Supplementary Table 4.

**Table 1:** Species, strain and sex distribution of animals in included studies.

Animal characteristic			Number of studies (%)
**Animal species**	Rat strain	Sprague-Dawley	5 (55.5%)
		Fisher 344/Brown Norwegian F1 hybrid	1 (11.1%)
	Mouse strain	C57BL/6	2 (22.2%)
		BALB/c	1 (11.1%)
**Animal sex**		Male	3 (33.3%)
		Female	4 (44.4%)
		Unspecified	2 (22.2%)

**Table 2:** Total numbers of animals included in each age group by each study.

Reference	Youngest	Middle	Oldest
Fenn et al. (33)	10–13 per group		10–13 per group
Roozbehi et al. (31)	12	12	12
Zhang et al. (34)	25		25
Kumamaru et al. (35)	5–7 per group		5–7 per group
Hooshmand et al. (28)	12		12
Siegenthaler et al. (29)	12	8	8
Siegenthaler et al. (32)	12	6	12
Genovese et al. (30)	60		60
Gwak et al. (36)	7	8	8
**Total** ^*^	155	34	152

^*^Calculated using lower value where studies report a range.

**Figure 2:**
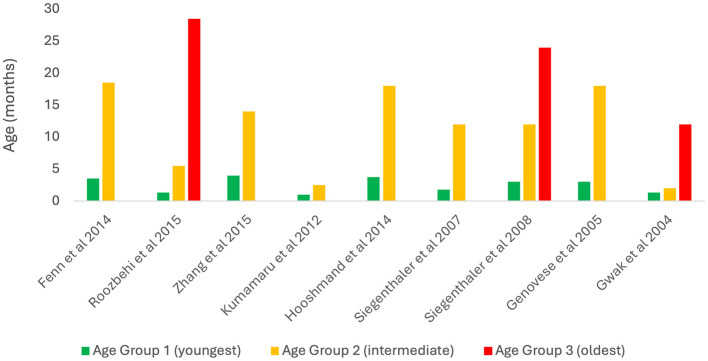
Ages of the different animal groups within included studies. Six studies used two groups (green and yellow bars, respectively) whilst the three remaining studies used three groups, with the oldest mice represented with a red bar.

All studies included at least two subgroups of ages (Figure 2). Age categories were adopted as reported in each study. Dichotomised young and aged animal subgroups were used in six studies (28–30, 33–35). Trichotomised age subgroups were used in three studies: 1) young, mature, and old (31), 2) young, aged, geriatric (32), 3) young, adult, middle aged (36). The youngest subgroup of animals across all studies had a mean age of 2.52 (SD 1.17) months, the middle subgroup had a mean age of 11.3 (SD 6.59) months, and the oldest had a mean age of 21.5 (SD 8.53) months.

All nine studies used traumatic models of SCI (Table 3). Spinal cord contusion using an Infinite Horizon impactor (32) was employed in six (6/9, 66.7%) studies (28, 29, 32–35), unilateral hemi-section using iridectomy scissors was used in two (2/9, 22.2%) studies (31, 36) and clip compression using aneurysm clips was used in one (1/9, 11.1%) study (30). A force of 200 kilodynes (kdyn) was used to generate contusion in three (3/9, 33.3%) studies (28, 29, 32), 75kdyn in one (1/9, 11.1%) study (33), 50kdyn in two (2/9, 22.2%) studies (34, 35). One (1/9, 11.1%) study used three groups of mice with different severities of contusion, generated using forces of 50kdyn, 70kdyn and 90kdyn (35). All SCI models involved thoracic injury between levels T8 and L1.

**Table 3:** Summary of preclinical SCI models categorized by injury type (54).

SCI model	Description	Relevant methods	Advantages	Limitations
Compression	Prolonged spinal cord compression	Aneurysm clip (30)	•Mimics fracture dislocations and burst fractures (36). •Relatively simple and inexpensive. •Can be used in different regions of the spinal cord. •Clips of varying closing force are available. •Incorporates a degree of vascular occlusion and ischaemia (36).	•Difficult to standardize due to variability in actual force applied and extent of cord compression (36). •Lacks acute impact phase of contusion models.
Contusion	Acute, blunt trauma to the spinal cord	Infinite Horizon impactor device to generate varying forces: •50kdyn (34, 35) •70kdyn (35) •75kdyn (33) •90kdyn (35) •200kdyn (28, 29, 32)	•Controlled method of inducing SCI with defined force (36). •Representative of human SCI (50). •Some devices allow real-time measurement of the force applied, allowing sub-optimal injuries to be excluded (36).	•Requires specialist impactor equipment. •Often severe animal disability post injury requiring significant care afterwards. •Risk of inconsistent injury due to difficulties stabilizing spinal cord during impact (36)
Transection (partial)	Partial cut of spinal cord.	Spinal cord hemisection Spinal cord hemisection (31, 36).	•Useful for investigating regeneration, degeneration and grafting (51). •Allows comparison of deficit and recovery between injured and healthy tracts in the same animal (36). •Consistent lesioning which can target specific anatomical tracts or regions.	•Pathophysiology may be less representative of human injuries which are most commonly contusive SCI (51). •May not accurately recapitulate all aspects of the pathophysiology of secondary injury.

### Locomotor outcome assessment

Locomotor outcomes were evaluated in all included studies (Table 4). The most commonly used measure of locomotor function was the Basso, Beattie, Bresnahan locomotor score (7/9 studies, 77.8%) (28–32, 35, 36). Other scoring systems used included the Basso Mouse Scale (2/9, 22.2%) (33, 34), CatWalk assessment (1/9, 11.1%) (28), Digigait assessment (1/9, 11.1%) (34), Footprint analysis (1/9, 11.1%) (35), four parameter kinematic analyses (1/9, 11.1%) (32) and GridWalk assessment (1/9, 11.1%) (34). Assessment time points ranged from one day post-injury to 56 days post-injury. Further information on the individual studies can be found in Supplementary Table 4.

**Table 4:** Summary of locomotor scoring tools assessing functional recovery post-SCI (54).

Scoring system	Summary
Basso, Beattie, Bresnahan Locomotor score (28–32, 35, 36)	Assesses hindlimb movement, paw placement, weight bearing, trunk stability, tail position, and limb coordination. Scored from 0 to 21; 0 is no hindlimb movement, 21 is normal function.
Basso Mouse Scale (33, 34)	Assesses the severity of SCI-induced paralysis based on hindlimb movement. Scored from 0 to 9.
Catwalk analysis (28)	An automated, observer-independent camera-based system for analysing gait and motor coordination in rodents. It captures detailed spatial and temporal parameters of movement (52, 53).
Digigait (34)	An objective locomotor analysis system that captures and digitizes footprints as animals walk on a transparent treadmill. Coordination, the ratio of forelimb-to-hindlimb stepping frequencies is calculated as the gait symmetry.
Footprint analysis (35)	Forelimbs and hindlimbs are dipped in green and red dyes, respectively. Animals are trained to walk on a paper covered narrow runway. A bright box is placed at the beginning of the runway, and a dark box is placed with their food at the end. Stride length is the distance from the start of a step with the rear paw through to the end of that step with the same paw. Stride width is defined as the distance from the left outermost hind paw digit to the right outermost hind paw digit.
Four parameter kinematic analyses (32)	Animals are videotaped using a camcorder from underneath 1cm marked grid lines. Videos are analyzed frame by frame and scored independently in a blinded manner for parameters including: •Rear paw stride length- distance from the start of a step with a rear paw through to the end of that step with the same paw •Stride width- distance from left outermost hind paw digit to the right outermost hind paw digit. •Toe spread – distance from the most lateral point of the lateral digit to the most medial point of the medial digit of each hind paw •Paw rotation – angle between axis of the rear paws with the baseline
GridWalk (34)	The grid walk is a horizontal ladder used to assess sensory-motor coordination. Animals traverse a horizontal ladder over rungs 4mm in diameter spaced 1.2cm apart from an open start platform to an enclosed goal box.

### Basso, Beattie, Bresnahan Locomotor Score

The BBB locomotor score was used to evaluate locomotor function in seven (7/9, 77.8%) studies ranging from one to 56 days post-injury (Table 5) (28–32, 35, 36). Across these studies, older animals consistently demonstrated slower and poorer locomotor recovery than younger animals following SCI.

**Table 5:** Studies using the BBB score to assess locomotor recovery following SCI.

Reference	Species	Age groups compared	Assessment period	Direction of effect^*^	Statistical significance
Gwak et al. (36)	Rat	Young, Adult, Middle-aged	Day 7–28	Young/Adult > Middle-aged	*p* < 0.05
Roozbehi et al. (31)	Rat	Young, Mature, Aged	Week 4–8	Young/Mature > Aged	*p* < 0.05
Genovese et al. (30)	Rat	Young, Older	Day 1–15	Young > Older	*p* < 0.05
Hooshmand et al. (28)	Rat	Young, Aged	Day 7–28	Young > Aged	*p* < 0.05
Kumamaru et al. (35)	Mouse	Young, Adult	Day 1–28	Young > Adult (Mild/Moderate contusion)	*p* < 0.05
Siegenthaler et al. (29)	Rat	Young, Aged, Geriatric	Week 1–4	Young > Aged/Geriatric	*p* < 0.01
Siegenthaler et al. (32)	Rat	Young, Aged (± Exercise)	Week 4–8	Young > Aged; Exercise > Sedentary	*p* < 0.05

^*^A > B denotes age-group A experienced greater improvement in the BBB score than age-group B.

Gwak et al. (36) identified no difference in BBB locomotor scores between 40-day old (young), 60-day old (adult) and 12-month-old (middle-age) rats prior to spinal cord hemisection, however spontaneous locomotor recovery occurred more rapidly in young and adult rats than in middle-aged rats. For example, on post-operative day seven the BBB scores in the young (11.29 ± 1.84) and adult (11.5 ± 2.06) group were significantly higher than the scores in middle-aged rats (1.63 ± 0.56; *p* < 0.05). This trend was observed throughout the testing period until day 28.

In addition, Roozbehi et al. (31) identified that 40-day old (young) and 5–6-month (mature) rats demonstrated a significant increase in the movement of their hindlimbs compared to 28–29-month (aged) rats between 4 weeks and 8 weeks post-injury. At 8 weeks post-injury the young and mature groups achieved BBB scores of 17 ± 1.47, and 16.8 ± 0.7, respectively, which were significantly higher than the BBB scores in the aged group (13.8 ± 1.63; *p* < 0.05).

Similarly, Genovese et al. (30) found that BBB locomotor scores in 3-month-old (young) rats were significantly higher than BBB motor scores in 18-month-old (older) rats from day 1 to day 15 following SCI. For example, at 15 days post–injury, young rats achieved BBB scores of approximately 11–12, whereas older rats remained significantly lower at around 6–7 (*p* < 0.05). In addition, Hooshmand et al. (28) reported that 18-month-old (aged)

female rats had significantly greater locomotor deficits compared to 15-week-old (young) female rats at seven days post-injury through to 28 days post-injury (*p* < 0.05). At 28 days post–injury, young rats achieved a BBB score of 15, whilst aged rats reached only 12.5, confirming a persistent and significant deficit in aged animals (28, 30). These findings demonstrated that aged animals had impaired gross locomotor recovery relative to younger counterparts across both early and subacute phases of SCI. The studies did not report measurements before day seven nor beyond day 28; it is unclear whether differences persisted or animals were culled at this time point.

Moreover, Kumamaru et al. (35) used three different forces to generate varying levels of spinal cord contusion in 4-week-old (young) and 10-week-old (adult) mice. Mild, moderate, and severe contusion was generated using forces of 50 kdyn, 70 kdyn and 90 kdyn, respectively. At 42 days post–injury, young mice achieved BBB scores of 14.8 ± 0.7 (mild), 10.2 ± 0.6 (moderate), and 2.9 ± 0.7 (severe), whereas adult mice scored 12.6 ± 0.4, 7.3 ± 0.4, and 2.1 ± 0.4, respectively. Significant differences persisted for mild and moderate injuries (*p* < 0.05).

Furthermore, using the BBB locomotor score Siegenthaler et al. (32) measured locomotor function in 3-month-old (young) rats, 12-month-old (aged) rats, 24-month-old (geriatric) rats. They found that aged and geriatric rats had poorer and delayed locomotor recovery compared to young rats. At 4 weeks post–injury, young rats achieved a mean BBB score of 13.2 ± 0.6, whereas aged and geriatric rats reached only 10.1 ± 0.7 and 8.9 ± 0.5, respectively (*p* < 0.01). The average weekly change in the BBB score was greatest for young animals after the 1 week post-injury and declined thereafter. By contrast the greatest change in BBB score for aged and geriatric animals was delayed until between two to three weeks post-injury (32).

In a further study, Siegenthaler et al. (29) explored the difference in BBB locomotor scores in 6–8-week-old (young) sedentary rats, 6–8-week-old exercised rats, 12-month-old (aged) sedentary rats and 12-month-old (aged) exercised rats over 8 weeks. At 4 weeks post–injury, young sedentary rats achieved a BBB score of 14.5 ± 0.4, significantly higher than both aged sedentary (10.8 ± 0.6) and aged exercised rats (12.3 ± 0.5) (*p* < 0.05). However, aged exercise animals demonstrated significantly greater locomotor function compared to aged sedentary rats at multiple timepoints post-injury. This study therefore demonstrated that the age-associated deficit in locomotor recovery following SCI can be partially mitigated with voluntary exercise.

### Basso Mouse Scale (BMS) Score

Two studies used the BMS score (33, 34), demonstrating that aged mice exhibited reduced and delayed locomotor recovery. Age alone was not found to influence locomotor function pre-injury, with baseline BMS scores not differing significantly between 14-month and 4-month-old animals (34). However, Zhang et al. (34) showed that functional recovery from SCI was significantly reduced in 14-month-old animals compared to 4-month-old animals with significant differences in BMS scores between three and 28-days post-injury. At 28 days post-injury, the 4-month-old animals had significantly improved and exhibited coordinated stepping, achieving a BMS score of 6.7 ± 0.5. The average BMS of 14-month-old mice was 5.0 ± 0.1, indicating the presence of some coordination (*p* < 0.05).

Similarly, Fenn et al. (33) showed that spontaneous recovery was significantly reduced in aged mice (18–19 months) when compared to adult mice (3–4 months) at all time-points post-SCI. Approximately 75% of the adult mice achieved a BMS score of five out of nine by 28 days post-injury but none of the aged mice reached this level of recovery.

### CatWalk

CatWalk gait analyses also indicated that older animals displayed greater locomotor impairment and instability after SCI than their younger counterparts. Hooshmand et al. (28) showed that aged rats (18 months) had a significant increase in walkway crossing time compared to young rats (15 weeks) following SCI, despite no differences at baseline. Furthermore, a significantly greater increase in base of support, corresponding to greater trunk instability, was seen in aged rats when compared to the younger group. These findings suggested decreased locomotor recovery in aged compared to younger rats (28).

### Digigait

Digigait assessments demonstrated that aging was associated with disrupted gait symmetry and poorer coordination following SCI. Zhang et al. (34) found that prior to SCI both 14-month-old mice and 4-month-old mice had a gait symmetry score of one indicating a one-to-one ratio between forelimb and hindlimb steps. At 27 days post-injury, gait symmetry scores for 4-month old mice were not significantly different from baseline but 14-month-old animals had significantly impaired gait symmetry compared to the 4-month old mice and compared to baseline.

### Footprint analysis

Footprint analysis further supported an age–related deficit, with older animals showing shorter stride length and greater paw rotation after injury. Using this tool at 42 days post-injury Kumamaru et al. (35) showed significantly improved functional outcomes in young mice (1 month) compared to adult mice (2.5 months), including stride length and paw rotation (35).

### Four-parameter kinematic analyses

Kinematic analyses revealed that aged and geriatric animals had altered gait parameters consistent with impaired locomotor recovery. Siegenthaler et al. (32) used four-parameter kinematic analyses to assess locomotor recovery post-SCI. They identified that 12-month-old rats (aged) and 24-month-old rats (geriatric) displayed a significantly greater stride width and shorter stride length than 3-month-old rats (young) at multiple time points following contusion SCI. The aged and geriatric rats displayed significantly greater digit spread and paw rotation at seven weeks following SCI compared to young rats, suggesting increased locomotor instability and poorer functional recovery (32).

### GridWalk

GridWalk testing found that older animals demonstrated poorer sensorimotor coordination after SCI. Zhang et al. (34) showed that following SCI 14-month-old mice had a significantly increased number of hindlimb foot slips on the Gridwalk horizontal ladder task when compared to 4-month-old mice.

### Risk of bias

It was unclear whether the allocation sequence was adequately generated in some studies. However, all studies reported that group locomotor characteristics were similar at baseline. Whether allocation was adequately concealed and whether animals were randomly housed was poorly reported.

One limitation in studying the impact of age in spinal cord injury (SCI) models is that animals' physical appearance may change over time, which can make allocation concealment challenging. Nevertheless, outcome assessor blinding was achieved in six studies. Four studies specified that animals were selected at random for outcome assessment, however it was mostly unclear whether caregivers or investigators were blinded.

Incomplete outcome data were adequately addressed across all studies. However, limited reporting of SYRCLE checklist items made it difficult to confidently determine overall risk of bias, which was therefore considered to be uncertain for all studies (Supplementary Table 2).

## Discussion

### Summary of main findings

Increasing age at the time of SCI is associated with poorer locomotor recovery. Across various SCI models, older animals demonstrated significantly lower locomotor scores compared to younger counterparts (28–30, 34, 37). Recovery trajectories differed by age, with younger animals showing the most rapid improvement whilst aged animals improved more slowly (37). Notably, one study showed that voluntary exercise before and after injury improved recovery in aged rats to rates comparable with young injured animals (29), suggesting the effects of age on recovery are potentially modifiable. Overall, the included studies support an age-dependent decline in recovery following SCI.

### Context of findings

Age-dependent decline in recovery appears to be multifactorial. Histological analyses from existing literature revealed that aged rodents exhibit greater lesion volumes, increased oedema, and greater apoptotic cell death following SCI (28–30). These structural differences are accompanied by immune cell infiltration, particularly of neutrophils and microglia/macrophages, at the injury site (28, 30, 33). However, given their focus on neurobehavioural outcomes measures, mechanistic evidence across included studies was heterogenous with only a subset providing histological analyses or interrogation of underlying molecular biology (33, 34).

The inflammatory response following SCI is known to play a dual role, initially contributing to secondary damage but later facilitating repair. In aged animals, this balance appears disrupted. Studies have shown that microglia in aged animals are more characteristically involved in sustained inflammation (30, 35). Moreover, aged macrophages demonstrate impaired phenotype switching, with reduced IL-10 expression, hindering the M2b anti-inflammatory phenotype, resulting in prolonged tissue damage and delayed recovery (34). Fenn et al. (33) reported reduced IL-1β mRNA expression in aged animals associated with fewer IL-4 receptor-positive microglia, limiting recruitment of macrophages.

Genovese et al. (30) suggested a mechanism by which microglia may contribute to sustained inflammation following spinal cord injury (SCI). Specifically, microglia in aged spinal cords produced more chemoattractant molecules, leading to increased neutrophil recruitment and exacerbated tissue damage compared to younger animals. Further investigation into age-related changes in microglial activation could clarify differences in immune responses and identify therapeutic targets. The same study also found that aged rats exhibited heightened vulnerability to SCI-induced oedema and inflammatory damage, likely due to impaired regulation of the inflammatory cascade. Moreover, they demonstrated that a robust antioxidant system mitigated secondary injury and supported motor recovery following compressional SCI (30).

Beyond immune dysregulation, age-related decline in neurotrophic support may also contribute to impaired recovery following SCI. Expression of BDNF and IGF-1, which promote neuronal survival and remyelination, are reduced in aged spinal cords (38, 39). Exercise has been shown to modulate these neurotrophins and attenuate oxidative stress, with Siegenthaler et al. (29) demonstrating that voluntary running before and after SCI improved locomotor outcomes in aged rats to levels comparable with their younger counterparts.

Taken together, these findings suggest that aging may alter both the structural and molecular landscape of SCI recovery. The convergence of increased lesion pathology, dysregulated inflammation, and diminished neurotrophic signaling underscores the need for age-specific therapeutic strategies. Further research is required to validate these mechanisms and explore their relevance to human SCI.

### Generalisability

All nine included studies employed rodent models, with no studies involving species more closely related to humans. This restricts translational relevance, as rodents differ significantly from humans in spinal cord anatomy, immune response, and recovery dynamics.

The SCI models used, contusion, hemisection and clip compression, each represent distinct pathophysiological mechanisms, none of which fully capture the pathobiology and heterogeneity of human SCI. Importantly, the studies did not encompass all forms of SCI seen clinically. In addition, the variability in injury mechanisms and forces employed in included studies also complicates direct comparison, although aged animals consistently demonstrated poorer recovery across all models. All lesions involved the thoracic spinal cord, which limits generalisability to cervical SCI, where functional recovery trajectories may differ (3).

Recovery timelines present another challenge. In humans, rehabilitation following SCI often spans months to years, with functional improvements continuing well beyond the acute phase. In contrast, the longest follow-up in the included studies was 56 days post-injury (40), which may therefore not adequately reflect the chronic recovery trajectory observed in clinical settings.

Age classification reflected additional heterogeneity. Terms such as “young,” “adult,” “aged,” and “mature” were used inconsistently across studies, with no standardized age groupings. This lack of uniformity makes it difficult to generalize findings across age groups or to map animal age categories to humans. Consensus on age stratification in preclinical research would enhance comparability and improve translational relevance (35).

### Limitations of included studies

This review synthesizes findings from nine preclinical studies, each investigating the impact of age on recovery following SCI. While the consistency of results across different injury models strengthens the overall conclusion that aged animals exhibit poorer locomotor recovery, the limited number of studies and the rodent nature of all studies reflects the currently narrow evidence base. This limitation restricts generalisability and understanding the clinical meaningfulness of findings to human SCI populations.

Another limitation lies in the heterogeneity of the included studies. Variability in SCI models, scoring systems and timing of functional assessments complicated direct comparison and precluded meta-analysis.

Risk of bias was difficult to assess. Although the SYRCLE risk of bias tool (26) was applied, allocation concealment and randomization are challenging in studies of this nature due to visible age-related differences between animals.

All included studies focused exclusively on traumatic SCI, with no representation of non-traumatic forms. This restricts generalisability, as non-traumatic SCI, such as those caused by degenerative or vascular processes, may involve distinct pathophysiological mechanisms and recovery trajectories (3, 41).

### Future directions

The consistent observation that aging impairs recovery following SCI in rodent models underscores the need for further translationally relevant research. Future studies should also include models that reflect degenerative and vascular SCI to allow for a broader understanding of age-related recovery mechanisms (41, 42).

Rodents, while offering a model, differ substantially from humans in spinal cord architecture, immune response, and recovery dynamics (43, 44). Larger animal models with closer anatomical and physiological resemblance to humans could improve the predictive value of therapeutic interventions (43). Additionally, recovery timelines in preclinical studies should be extended beyond the typical 56-day window to better reflect the prolonged rehabilitation seen in clinical practice (45).

Mechanistic studies should also be prioritized, particularly those exploring microglial phenotype switching, neutrophil recruitment, and neurotrophic factor expression in aged spinal cords (29, 30, 35, 38). These pathways offer promising therapeutic targets but remain incompletely understood.

Compounds with postulated anti-inflammatory or neuroprotective potential, such as astaxanthin (46) and polyphenols in green tea extract (47), should be rigorously evaluated in age-stratified models, with attention to dosing, toxicity, and delivery routes. Non-pharmacological interventions such as exercise also warrant further investigation, given their capacity to modulate neurotrophic support and oxidative stress (48, 49).

Improving methodological transparency, reducing bias, and broadening the scope of included studies will also be vital to building robust evidence base to inform age-tailored interventions for SCI. A consensus-based process would be valuable to agree standardized reporting guidelines for this field.

## Conclusions

The results of included studies demonstrate that increasing age at the time of SCI is associated with poorer locomotor recovery across a range of preclinical models of traumatic SCI. This relationship was reversible by exercise in a single study. However, the results should be interpreted in the context of unclear risk of bias of included studies. This emphasizes the need for additional studies with consistent methodologies, age stratification and outcome reporting at standardized time-points to evaluate the effect of age on locomotor recovery following SCI. Furthermore, the potential interactions between age and sex in SCI recovery trajectories is another avenue for future research, given the known sex differences in the neuroinflammatory responses.

## Data Availability

The original contributions presented in the study are included in the article/Supplementary material, further inquiries can be directed to the corresponding authors.
