# Supplementary material for: The impact of aging on locomotor recovery in preclinical models of traumatic spinal cord injury: a systematic review
Source: Front Neurol. 2026 Jun 1;17:1745250. doi: 10.3389/fneur.2026.1745250 (PMC13267688; doi:10.3389/fneur.2026.1745250)
Supplement: Supplementary file 1 [file Table_1.docx]

**Supplementary Table 1.** Search strategy in MEDLINE and Embase are shown,

| Database | No | Search Query | Results |
| --- | --- | --- | --- |
| MEDLINE | | | |
|  | #1 | (("nervous system disease"[MeSH Terms] OR "nerve degeneration"[MeSH Terms] OR "spinal diseases"[MeSH Terms] OR "spinal cord injuries"[MeSH Terms] OR "spinal cord ischemia"[MeSH Terms] OR "central cord syndrome"[MeSH Terms] OR "spinal cord injury"[MeSH Terms] OR "neurotrauma"[Title/Abstract] OR "SCI"[Title/Abstract] OR ((neuro* OR brain* OR nerve*) AND (trauma* OR demyelinat* OR remyelinat*))[Title/Abstract] OR (myelopathy AND (traumatic OR post-traumatic))[Title/Abstract] OR ((spine OR spinal) AND (fracture* OR wound* OR trauma* OR injur* OR damag*))[Title/Abstract] OR (spinal cord AND (contusion OR laceration OR transaction OR trauma OR ischemia))[Title/Abstract] OR "central cord injury syndrome"[Title/Abstract] OR "central spinal cord syndrome"[Title/Abstract])  AND  ("regeneration"[MeSH Terms] OR "regenerative capacity"[Title/Abstract] OR "neuroregeneration"[Title/Abstract] OR "nerve regeneration"[MeSH Terms] OR "cell regeneration"[MeSH Terms] OR "nerve fiber regeneration"[MeSH Terms])  AND  ("aging"[MeSH Terms] OR "ageing"[MeSH Terms] OR "age factors"[MeSH Terms] OR "aging"[Title/Abstract] OR "age-related"[Title/Abstract] OR "age factor*"[Title/Abstract] OR ((aging OR ageing OR age) AND (related OR associated OR dependent))[Title/Abstract]))  AND  ((excludepreprints[Filter] OR medline[Filter]) AND (casereports[Filter] OR classicalarticle[Filter] OR clinicalstudy[Filter] OR clinicaltrial[Filter] OR multicenterstudy[Filter] OR observationalstudy[Filter] OR randomizedcontrolledtrial[Filter] OR technicalreport[Filter]) AND (humans[Filter]) AND (female[Filter] OR male[Filter]) AND (english[Filter])) | 1989 |
| Embase | | | |
|  | #1 | #1 ('nervous system disease'/exp OR 'nerve degeneration'/exp OR 'spinal diseases'/exp OR 'spinal cord injuries'/exp OR 'spinal cord ischemia'/exp OR 'central cord syndrome'/exp OR 'cervical spine injury'/exp OR 'spine injury'/exp OR 'spinal cord injury'/exp OR 'neurotrauma*':ti,ab OR 'SCI':ti,ab OR ((neuro* OR brain* OR nerve*) NEAR/4 (trauma* OR demyelinat* OR remyelinat*)):ti,ab OR (myelopathy NEAR/3 (traumatic OR post-traumatic)):ti,ab OR ((spine OR spinal) NEAR/3 (fracture* OR wound* OR trauma* OR injur* OR damag*)):ti,ab OR (spinal cord NEAR/3 (contusion OR laceration OR transaction OR trauma OR ischemia)):ti,ab OR 'central cord injury syndrome':ti,ab OR 'central spinal cord syndrome':ti,ab)  #2 ('regeneration'/exp OR 'regeneration':ti,ab OR 'regenerative capacity':ti,ab OR 'neuroregenerat*':ti,ab OR 'nerve regeneration'/exp OR 'nerve regenerat*':ti,ab OR 'cell regeneration'/exp OR 'cell regenerat*':ti,ab OR 'nerve fiber regeneration'/exp OR 'nerve fibre regenerat*':ti,ab)  #3 ('aging'/exp OR 'ageing'/exp OR 'age factors'/exp OR 'aging':ti,ab OR 'ageing':ti,ab OR 'age-related':ti,ab OR 'age factor*':ti,ab OR ((aging OR ageing OR age) NEAR/3 (related OR associate* OR dependent)):ti,ab)  #4 #1 AND #2 AND #3 AND [english]/lim AND [humans]/lim AND [article]/it | 1730 |
