# Supplementary material for: The impact of aging on locomotor recovery in preclinical models of traumatic spinal cord injury: a systematic review
Source: Front Neurol. 2026 Jun 1;17:1745250. doi: 10.3389/fneur.2026.1745250 (PMC13267688; doi:10.3389/fneur.2026.1745250)
Supplement: Supplementary file 2 [file Table_2.docx]

**Supplementary Table 2:** SYRCLE risk of bias assessment.

The Systematic Review Center for Laboratory Animal Experimentation (SYRCLE) Tool28 was used to evaluate risk of bias.

Abbreviations: NS = not stated

| Bias assessment question | A.Fenn et al 2014 | A.Roozbehi et al 2015 | B.Zhang et al 2015 | H.Kumamaru et al 2012 | M.Hooshmand et al 2014 | M.Siegenthaler et al 2008 | M.Siegenthaler et al 2008 | T.Genovese et al 2005 | Y.Gwak et al 2004 |
| --- | --- | --- | --- | --- | --- | --- | --- | --- | --- |
| Was the allocation sequence adequately generated and applied? | Not Stated | Not Stated | Not Stated | Not Stated | Not Stated | Not Stated | Not Stated | Not Stated | Not Stated |
| Were the groups similar at baseline or were they adjusted for confounders in the analysis? | Yes | Yes | Yes | Yes | Yes | Yes | Yes | Yes | Yes |
| Was the allocation adequately concealed? | Not Stated | Not Stated | Not Stated | Not Stated | Not Stated | Not Stated | Not Stated | Not Stated | Not Stated |
| Were the animals randomly housed during the experiment? | Yes | Yes | Yes | Not Stated | Not Stated | Yes | Not Stated | Not Stated | Not Stated |
| Were the caregivers and/or investigators blinded from knowledge which intervention each animal received during the experiment? | Yes | Not Stated | Not Stated | Not Stated | Not Stated | Not Stated | Not Stated | Not Stated | Not Stated |
| Were animals selected at random for outcome assessment? | Not Stated | Not Stated | Not Stated | Not Stated | Not Stated | Not Stated | Not Stated | Not Stated | Not Stated |
| Was the outcome assessor blinded? | Yes | Yes | Not Stated | Yes | Yes | Not Stated | Yes | Yes | Not Stated |
| Were incomplete outcome data adequately addressed? If there wasn’t any incomplete outcome data I’ve stated ‘yes’. | Yes | Yes | Yes | Yes | Yes | Yes | Yes | Yes | Yes |
| Are reports of the study free of selective outcome reporting? | Yes | Yes | Yes | Yes | Yes | Yes | Yes | Yes | Yes |
| Was the study apparently free of other problems that could result in high risk of bias? | Yes | Yes | Yes | Yes | Yes | Yes | Yes | Yes | Yes |
