# Supplementary material for: The impact of aging on locomotor recovery in preclinical models of traumatic spinal cord injury: a systematic review
Source: Front Neurol. 2026 Jun 1;17:1745250. doi: 10.3389/fneur.2026.1745250 (PMC13267688; doi:10.3389/fneur.2026.1745250)
Supplement: Supplementary file 4 [file Table_4.docx]

**Supplementary table 4:** data extraction table.

| **Author (Year /Country)** | **Article Title** | **Sample Features**  **(species, gender)** | **Ages** | **Injury Model** | **Interventions / grouping** | **Outcome Assessed** | **Time of Assessment** | **Statistical Analysis** | **Main Conclusions** |
| --- | --- | --- | --- | --- | --- | --- | --- | --- | --- |
| Fenn et al., 2014  USA | IL-4 Signaling Drives a Unique Arginase /IL-1 Microglia Phenotype and Recruits Macrophages to the Inflammatory CNS: Consequences of Age-Related Deficits in IL-4R after Traumatic Spinal Cord Injury | **Species:** BALB/c mice | Adult (3-4 months)    Aged (18-19month) | Contusion using Infinite Horizons device    75kdyn  SCI level: T9 | Adults and Aged Controls: laminectomy    Adult and Aged SCI group: laminectomy followed by moderate SCI contusion injury (75kdyn). | Basso Mouse Scale (BMS) | 1,3,5,7,14,21 and 28 days post-injury (dpi) | ANOVA  One way (pretreatment and treatment), two way (pretreatment x treatment) or three-way (pretreatment x treatment x time) ANOVA      Differences between treatment means– F-protected t test    Particular BMS score evaluated by the X^2 test.    Means+-SEM p<0.05 and tendency p</0.1 | - Age does not affect BMS score for uninjured mice as controls had similar scores over the 28 day time period.  Controls (n=4) - Spontaneous recovery was significantly reduced in aged mice compared with adult mice at 1,3,4,7,21 and 28 dpi in aged mice compared with adult mice (Age X SCI: F(1,153) =12.96, p<0.0001. - Approximately 75% of the adult mice achieved a BMS score of 5 by 28dpi but none of the aged SCI reached this level of recovery (p<0.0001). SCI groups (n=10-13) |
| Roozbehi et al., 2015  Iran | Age-Associated Changes on Axonal Regeneration and Functional  Outcome after Spinal Cord Injury in Rats | **Species:** Sprague-Dawley Rats    **Sex:** male | Young (40days) (n=12)    Mature (5-6 months) (n=12)    Old (28-29months (n=12)) | Spinal **Hemi section** at L1 level using iridectomy scissors to disrupt major unilateral descending pathways | Young (40 days): 12 animals  Mature (5-6 months): 12 animals  Old (28-29 months):12 animals | Basso,Bresnahan and Beattie (BBB) | One dpi and weekly for 8 weeks after lesion | Mean ± SD  One way ANOVA used for data analysis, Tukey test for post hoc analysis    Differences considered significant at P<0.05 | - BBB scores increased at the end of three weeks post-surgery for each of the three groups (mean ± SD): 12.85 ± 1.28, 1.02 ± 0.43 and 11.92 ± 1.61) for young, mature, and old groups, respectively - From 4^th^ week until the 8^th^ the young and mature rats showed a significant increase in movements of their hindlimbs compared to the aged group (p<0.05) - The young group showed consistent plantar stepping and consistent forelimb-hindlimb (FL-HL) coordination, whereas the old rats demonstrated limited FL-HL coordination. - At the end of eight weeks, young and mature animals achieved a plateau score of (mean+-SD) 17 ± 1.47, and 16.8 ± 0.7 respectively and the old rats reached an average score of 13.8 ± 1.63. Significant difference between young and mature groups compared to the age group during week 4 to eight (p<0.05) |
| Zhang et al., 2015  USA | Age decreases macrophage IL-10 expression: Implications for functional  recovery and tissue repair in spinal cord injury | **Species:** C57BL/6 mice    **Sex:** Female | 4-month-old (n=25),  14-month-old (n=25) | Contusion using Infinite Horizons device    50 kdyn  SCI level: T9 | 4 month old group (n=7 for behavioral analysis)  14 month old group (n=7 for behavioral analysis) | Basso Mouse Scale (BMS)    Digigait to assess forelimb and hindlimb coordination    Gridwalk | BMS: 1,3,7,14,21,28 dpi by two trained observers    Digigait: 27dpi      Gridwalk: 27dpi | One- or two-way ANOVA followed by Tukey’s, or Dunnett’s test for multiple comparisons.    Independent sample t-tests were used where appropriate      *p* ≤ 0.05 | BMS:   - Age alone does not influence function since baseline scores for all outcome measures did not differ between 14 and 4 MO animals. - Following SCI both age groups had significant locomotor impairments that improved over time (time factor, p<0.0001) as measured by the BMS score - Recovery was significantly reduced in 14 vs 4 MO animals (age x time interaction p<0.001). Differences in BMS scores were significant by 3dpi and lasted throughout the 28dpi survival period (p<0.05) - At 28dpi, 4MO animals significantly improved recovery over time with coordinated stepping and reached a BMS score of 6.7 ± 0.5 whereas average BMS of 14MO mice was 5.0 ± 0.1 with some coordination. (2-way ANOVA time x age interaction p=0.02; main effect of age= 0.004 - Coordination differences at 28dpi in 14vs 4 MO animals with average BMS subscores – 28dpi in 14vs 4MO animals (average BMS subscores 1.3 ± 0 and 6.7 ± 1.3 for 14 and 4 MO animals respectively)     Digigait:   - Prior to SCI both 14 and 4 MO had a gait symmetry score of 1 indicating a 1:1 ratio between fore and hindlimb steps (p>0.2 for baseline 4 vs 14MO) - 14 MO had significantly impaired gait symmetry as compared to 4MO SCI and baseline groups (p<0.05 vs all other groups) whereas 4MO were not significantly different from baseline 27dpi     Gridwalk:   - 4 weeks post injury 14MO had increased hindlimb foot slips on the Gridwalk task compared to 4MO animals (p=0.05) |
| Kumamaru et al., 2012  Japan | Age-Related Differences in Cellular and Molecular Profiles of Inflammatory Responses After Spinal Cord Injury | **Species:** C57BL/6 mice    **Sex:** Female | Young (4-week-old, bodyweight 17-19g)    Adult (10-week-old, 18-21g) | **Contusions** using Infinite Horizons device  SCI: T9 | Different contusion severities  Mild 50kdyn, Moderate 70kdyn, Severe 90kdyn) | BBB    Footprint analysis (stride length, stride width, paw rotation) | 7,14,21,28,35,42 dpi | Mann-Whitney U-test or Student’s t test. P<0.05 as significance. Mean+-SEM | - Young mice showed better and more rapid functional recovery from the acute phase of SCI than adult mice, regardless of the injury and significant differences were retained until the end point of evaluation in mild and moderate injuries. - The BBB scores of both groups at 42 days after SCI was 14.8 ± 0.7 and 12.6 ± 0.4, 10.2 ± 0.6 and 7.3 ± 0.4, 2.9 ± 0.7 and 2.1 ± 0.4 for mild, moderate, and severe injuries, respectively. - In addition, footprint analysis at 42 d after SCI also revealed that improved functional outcomes in young mice in terms of the stride length and paw rotation than those in adult mice.      - BBB revealed a significantly improved functional recovery in young than that in adult mice irrespective of the severity of the injury, especially after mild and moderate injuries.      - Footprint analyses of the young and adult mice at 42d after SCI. The forelimb and hindlimb stride pattern showed improved functional outcomes in young mice compared to that in adult mice (n= 5-7 each group) p<0.05, Students test represented as mean+/SEM |
| Hooshmand et al., 2014  USA | Characterization of recovery, repair, and  inflammatory processes following contusion  spinal cord injury in old female rats: is age a  limitation? | **Species:** Fisher 344/Brown Norwegian F1 hybrid rats    **Sex:** Female | Young (15 weeks old)  n=12    Aged (18 months)  n=12 | **Contusion** using Infinite Horizons device    200 kdyn  SCI level: T9 | Young (15 weeks old)  N=12    Aged (18 months)  N=12 | BBB  Catwalk analysis   - BBB open field locomotor scale | BBB:  Prior to injury, 2,7 dpi and weekly thereafter for 4 weeks    CatWalk analyses:  Prior to injury and terminally at 28dpi | Repeated measures ANOVA demonstrated a significant difference between young versus aged animals    Comparisons between groups on BBB locomotor scale – repeated measures ANOVA with post-hoc Bonferroni Dunn    Student’s t-tests were used for group comparisons of data obtained from CatWalk. CH50 assays    Values of p</ 0.05 = significant all data expressed as mean +-SEM | BBB:   - Aged rats greater locomotor deficits compared to young at 7dpi lasting through 28dpi - Significant difference in BBB scores 7,21 and 28 dpi. - 28dpi: Young score 15 and aged 12.5 (significant difference) gross locomotor recovery in aged animals significantly impaired relative to young.     CatWalk analyses:   - Significant increase in crossing time of aged animals post injury. - Increase in contact area in aged animals post injury vs young animals post injury – impairment in movement kinetics. - Injured young showed decreased contact area relative to their pre-injury baseline. - may represent avoidance of complete placement due to altered sensitivity. - Greater increase in base of support post injury seen in aged vs younger animals – significantly wider base of support comp to young suggesting compensation for trunk instability. - Increased walkway crossing time and base of support suggest decreased locomotor recovery in aged rats. |
| Siegenthaler et al., 2008a  USA | Myelin pathogenesis and functional deficits following SCI are age-associated | **Species:** Sprague-Dawley rats  **Sex:** Female | Young (3 months; 200-220g),    Aged (12months; 250-450g)      Geriatric (24months; 430-480g) | **Contusion** using Infinite Horizons device    200 kdyn  SCI level: T10 | SCI and measurement of outcomes in young, aged and geriatric rats | Basso, Beattie, Bresnahan     Four-parameter kinematic analyses, | Before injury, 2 weeks post injury for young animals, 4 weeks post-injury for aged and geriatric animals | BBB scores analyzed by multivariate repeated measures ANOVA to assess significance between each group over time and by t-test for significant at each time point    Kinematic analyses: t-test used to determine difference between groups | - Aged and geriatric rats have a delayed rate of locomotor recovery following contusion SCI compared to young rats. - Young animals demonstrated significantly greater p<0.01 locomotor capabilities post injury as compared to aged and geriatric animals. - No sig difference between locomotor capabilities between aged and geriatric animals (p>0.05) - Average change in BBB score for 1 week was greatest in young animals within first week and declined thereafter - Greatest change in BBB score for aged and geriatric animals, was delayed until the second week after which the rate of recovery decreased. - Change in BBB score between 1 week and 2-week post injury was significantly greater in young animals (p<0.01)     Four-Parameter Kinematic Locomotor recovery   - Aged and geriatric animals had a significantly higher stride width than young animals at multiple time points following contusion SCI (p<0.001) - Aged and geriatric animals had a significantly shorter stride length at multiple time points following contusion SCI (p<0.05) - Aged animals had significantly greater digit spread at 4- and 7-weeks following contusion SCI (p<0.05) - Aged and geriatric animals had significantly greater paw rotation at 4,5,6 and 7 weeks post contusion SCI (p<0.05)       Aged and geriatric have significantly greater area of pathology and amount of demyelination + less remyelination compared to young rats following contusion SCI  Age associated decline in rate and extent of locomotor and bladder recovery following contusion SCI |
| Siegenthaler et al., 2008b  USA | Voluntary running attenuates age-related deficits following SCI | **Species:** Sprague Dawley rats | Aged adult (n=12; 600-800g; 12-month-old)      Young adult animals (n=12)  350-500g; 6-8 weeks old | **Contusion** using Infinite Horizons device    200 kdyn  SCI level: T8-11 | Examined the effect of voluntary exercise (running wheel), pre- and post-injury, on locomotor recovery and myelin pathology following contusion SCI | BBB | Pre-injury and weekly post injury for 8 weeks | BBB scores analyzed by chi-squared test at each time point (weekly post injury for 8 weeks)    Expected range- values from the young group when testing for significance with aged sedentary group      Values from aged sedentary group as expected range when testing for significance with the aged exercise group | Paper found: Rate of locomotor recovery following SCI is significantly delayed in aged rats as compared to young rats and is associated with a greater degree of pathology and demyelination. Age-related delay in locomotor recovery following SCI & age-related increased in histopathology following SCI. Exercise attenuates age related locomotor and histopathological deficits following SCI.     - Finding: exercise improves locomotor recovery of injured aged rats such that it is comparable to the recovery rate of injured young rats: decreased area of pathology and amount of demyelination. – voluntary wheel running - Rate of locomotor recovery and myelin pathology in aged exercised rats similar to that of young sedentary rats after injury - exercise attenuated the delayed recovery of function and associated histopathology in aged rats     Locomotor recovery Significant findings:   - Aged exercise animals demonstrated significantly greater (p<0.05) locomotor capabilities at multiple times post-injury as compared to aged sedentary animals - Significant differences between aged sedentary animals and the young or aged exercise animals at 2,3,4,7 and 8 weeks post injury – age-associated deficit in locomotor recovery that can be significantly improved with voluntary wheel running - Young animals demonstrated significantly greater (p<0.05) locomotor capabilities at 4 weeks post-injury compared to sedentary aged and exercise aged groups. - Average change in BBB score during one week was greatest in young and aged exercise animals within the first week, gradually declining over the next couple weeks. - The greatest change in BBB score for aged sedentary animals was delayed until the second week after which recovery decreases. – peak locomotor recovery delayed by one week in aged sedentary group     Interesting point: exercise influences outcome of CNS injury.  1) physical activity increases levels of IGF-1 and BDNF which are important in oligodendrocyte and neuron cell survival  2) improve locomotor function following SCI  3) decrease susceptibility to free radical damage which has implications for neuroprotection    i.e. Increase factors associated with oligodendrocyte and neuron cell survival, improve locomotor function and have a neuroprotective effect |
| Genovese et al., 2005  Italy | Increased oxidative-related mechanisms in the spinal cord injury in old rats | **Species:** Sprague-Dawley rats  **Sex:** Male | Old-SCI- operated rat group; 18 months (n=30)    Young SCI- operated; 3 months rat group (n=30)    Old sham group same procedure but no aneurysm clip applied (n=30)    Young sham group identical to old sham group (n=30) | **Clip compression** SCI with aneurysm clip  50 g of force  SCI level: T5-T8 | Behavioural responses following traumatic SCI | BBB | 24h after SCI animals (n=10) sacrificed (molecular analysis)      Test for functional deficit at 1 day until 15^th^ day BBB score | Mean (S.E.M)    One-way ANOVA followed by a Bonferroni’s post hoc test for multiple comparisons  P<0.05 = significant | Interesting points: SCI in old rats, severe trauma characterized by edema and neutrophil infiltration. Immunohistochemical examination – increase in immunoreactivity for nitro tyrosine.  Young rats degree of a) spinal cord inflammation and tissue injury (histological score), b) nitrotyrosine, c) PARS and d) neutrophil infiltration markedly reduced in spinal cord tissue obtained from young rats.       - Ageing significantly worsened recovery of limb function and caused increase mortality rate when compared with young rats. - The BBB motor score of SCI-Young rats was measured to be significantly greater than the BBB motor score of SCI-Older rats on everyday post injury. |
| Gwak et al., 2004  USA | Effect of Age at Time of Spinal Cord Injury on Behavioral Outcomes in Rat | **Species:** Sprague-Dawley rats  **Sex:** Male | Young (164.6± 2.4g, 40 days n=7),    Adult (273.3 ± 2.9g, 60 days n=8)    Middle-age (546 ± 4.3g, 12 months, n=8) | Unilateral **Hemi section** of spinal cord at T13 | Behavioral responses to mechanical and thermal stimuli following SCI | BBB    Open field tests, paw withdrawal frequency (PWF), & paw withdrawal latency (PWL) t | Before injury and post operated days 1,7,14,21 and 28 days) | One way or two-way analysis of variance with repeated measures with time as a factor followed by the Duncan’s test for multiple comparison, using the SAS program (version 8.0). *p* < 0.05)  Mean ± standard error (mean ± SE) | BBB   - Locomotor scores were the same prior to hemisection in all three groups (21/21). - Significantly higher scores were achieved more rapidly in young and adult groups compared to middle-age group, at 1.63+-0.56 (*p* < 0.05) and lasted the entire test period to post operative day 28. - On Post operative day 7, the BBB scores in the young and adult groups were 11.29 ± 1.84 and 11.5 ± 2.06, respectively. - Spontaneous locomotor recovery occurred more rapidly in young and adult than in middle-aged rats. |
